# Assessment of linear regression of peripapillary optical coherence tomography retinal nerve fiber layer measurements to forecast glacuoma trajectory

**DOI:** 10.1371/journal.pone.0296674

**Published:** 2024-01-12

**Authors:** Chris Bradley, Kaihua Hou, Patrick Herbert, Mathias Unberath, Greg Hager, Michael V. Boland, Pradeep Ramulu, Jithin Yohannan

**Affiliations:** 1 Wilmer Eye Institute, Johns Hopkins University School of Medicine, Baltimore, Maryland, United States of America; 2 Malone Center of Engineering in Healthcare, Johns Hopkins University School of Medicine, Baltimore, Maryland, United States of America; 3 Massachusetts Eye and Ear, Harvard Medical School, Boston, Massachusetts, United States of America; Aravind Eye Hospital, INDIA

## Abstract

Linear regression of optical coherence tomography measurements of peripapillary retinal nerve fiber layer thickness is often used to detect glaucoma progression and forecast future disease course. However, current measurement frequencies suggest that clinicians often apply linear regression to a relatively small number of measurements (e.g., less than a handful). In this study, we estimate the accuracy of linear regression in predicting the next reliable measurement of average retinal nerve fiber layer thickness using Zeiss Cirrus optical coherence tomography measurements of average retinal nerve fiber layer thickness from a sample of 6,471 eyes with glaucoma or glaucoma-suspect status. Linear regression is compared to two null models: no glaucoma worsening, and worsening due to aging. Linear regression on the first M ≥ 2 measurements was significantly worse at predicting a reliable M+1^st^ measurement for 2 ≤ M ≤ 6. This range was reduced to 2 ≤ M ≤ 5 when retinal nerve fiber layer thickness measurements were first “corrected” for scan quality. Simulations based on measurement frequencies in our sample—on average 393 ± 190 days between consecutive measurements—show that linear regression outperforms both null models when M ≥ 5 and the goal is to forecast moderate (75^th^ percentile) worsening, and when M ≥ 3 for rapid (90^th^ percentile) worsening. If linear regression is used to assess disease trajectory with a small number of measurements over short time periods (e.g., 1–2 years), as is often the case in clinical practice, the number of optical coherence tomography examinations needs to be increased.

## Introduction

Glaucoma is an eye disease that causes damage to the optic nerve and is the leading cause of irreversible blindness [1]. Because the optic nerve consists of retinal ganglion cell axons that traverse the retina through the retinal nerve fiber layer (RNFL), measurements of RNFL thickness can be used as an indirect measure of optic nerve damage. Clinicians often use linear regression on optical coherence tomography (OCT) measurements of peripapillary RNFL thickness to detect glaucoma worsening and forecast future disease course with current treatment [2–4].

The accuracy of linear regression depends on the accuracy and reliability of OCT RNFL thickness measurements. Prior studies have estimated the percentage of errors in retinal boundary detection algorithms used by Zeiss Cirrus OCT (the instrument used in this study) and also measured its test-retest reliability [5–8]. Other studies have assessed the effect of scan quality metrics such as signal strength on RNFL thickness measurements, suggesting that OCT measurements can be “scan quality corrected”—reliability can be increased post-hoc given knowledge of OCT signal strength and glaucoma severity [9].

The accuracy of linear regression also depends on the distribution of rates of RNFL thickness change in the patient population, as was shown in a study [10] that estimated the accuracy of detecting moderate and rapid glaucoma worsening to be less than 50% given the relatively low OCT examination frequencies in clinical settings—once every 390 ± 186 days in a database of over 12,000 eyes with glaucoma or glaucoma suspect status. The small number of measurements typically used in clinical settings to assess RNFL thickness worsening raises the question of whether linear regression significantly outperforms a null model (e.g., a model predicting no glaucoma worsening, or a model predicting worsening due to aging). This is important because clinicians use linear regression of OCT RNFL thickness measurements to make decisions on the continuation or escalation of therapy, and erroneous decisions may result if future RNFL thickness worsening is predicted due to measurement error (i.e., small sample size).

In this study, we investigate the effect of different numbers of RNFL measurements over time on the ability of linear regression to accurately forecast future RNFL thickness worsening using data from a large sample of glaucoma and glaucoma suspect patients. Linear regression is compared to “no worsening” and “aging” null models. We also assess whether including only reliable RNFL measurements (those with higher signal strength) or correcting RNFL thickness measurements for low signal strength before applying linear regression improves performance. Since the true rate (the rate without OCT measurement error) of RNFL thickness worsening is unknown, we compare model abilities to predict future reliable measurements of OCT RNFL thickness.

## Materials and methods

### Forecasting models

We compared the performance of 5 different models to predict a reliable M+1^st^ Zeiss Cirrus OCT measurement of average RNFL thickness from the first M ≥ 2 measurements. We defined a “reliable” OCT scan as having signal strength (SS) of 8 or greater (higher signal strength is associated with higher OCT measurement reliability [11]) and an RNFL thickness above the measurement floor of 57 μm for Cirrus OCT [12, 13]. Two null models were tested. The “no worsening” null model predicted the M+1^st^ reliable measurement to be the mean of the first M measurements, while the “aging” null model predicted the M+1^st^ reliable measurement using the best-fit line to the first M measurements with a fixed slope of 0.365 μm/yr—the rate of average RNFL thickness loss over time measured in 121 healthy eyes in a recent study [14] using Cirrus OCT.

Three linear models were tested, the first being simple linear regression without any cutoffs or adjustment for poor SS values. The second linear model was “SS-filtered” in that linear regression was applied only to measurements with SS ≥ 6 (i.e., to a subset of more reliable measurements). The third linear model applied linear regression to “scan quality corrected” OCT RNFL thickness measurements based on results from a previous study (see Table 1) [15]. Scan quality correction uses SS and glaucoma severity, with glaucoma severity defined using mean deviation (MD): mild (MD > –6 dB), moderate (–6 dB ≥ MD > –12 dB), and severe (MD ≥ –12 dB). Scan quality correction was only applied to the first M measurements, not to the predicted M+1^st^ measurement. For all models, we used mean absolute error (MAE) to quantify accuracy for predicting a reliable M+1^st^ measurement. The Wilcoxon rank sum test was used to determine whether the error distributions between any two models were significantly different.

**Table 1:** Scan quality correction.

Mild Glaucoma	
3 ≤ *SS*	*R*_*corrected*_ = 0.67*(10 –*SS*)+*R*_*measured*_
*SS* < 3	*R*_*corrected*_ = 16.34*(3 –*SS*)+0.67*(10–*SS*)+*R*_*measured*_
Moderate Glaucoma	
3 ≤ *SS*	*R*_*corrected*_ = 0.75*(10 –*SS*)+*R*_*measured*_
*SS* < 3	*R*_*corrected*_ = 15.7*(3 –*SS*)+0.75*(10–*SS*)+*R*_*measured*_
Severe Glaucoma	
3 ≤ *SS*	*R*_*corrected*_ = 1.25*(10 –*SS*)+*R*_*measured*_
*SS* < 3	*R*_*corrected*_ = 16.03*(3 –*SS*)+1.25*(10–*SS*)+*R*_*measured*_

Relation between measured RNFL thickness (*R*_*measured*_) and scan quality corrected RNFL thickness (*R*_*corrected*_) given signal strength (*SS*). Results are from a previous study [15].

### Data collection

The study protocol was approved by the Johns Hopkins University School of Medicine institutional review board and adhered to the Declaration of Helsinki. Data were collected from patients 18 years or older with glaucoma or glaucoma suspect status who were recruited at the Wilmer Eye Institute from April 2013 to July 2021. Data analysis for the current study was performed starting from August 2021 using only deidentified data with individual patient identification information masked to the authors. Based on the previous discussion, eyes had to satisfy 4 inclusion criteria. At least one reliable (SS ≥ 8) M+1^st^ measurement of RNFL thickness was required for M ≥ 2. To apply linear regression, each eye had to have at least 3 OCT measurements of RNFL thickness from different visits. The SS-filtered linear model required that at least two of the first M measurements had SS ≥ 6. OCT scan quality correction required eyes to have at least one reliable VF within two years of the first OCT visit—scan quality correction requires both OCT SS and glaucoma severity defined by VF MD—with a reliable VF being defined as any VF with less than 15% false positives, and either less than 25% false negatives for mild or moderate glaucoma (MD > –12) or less than 50% false negatives for severe glaucoma (MD ≤ –12) [15].

### Simulations

**Fig 1:**
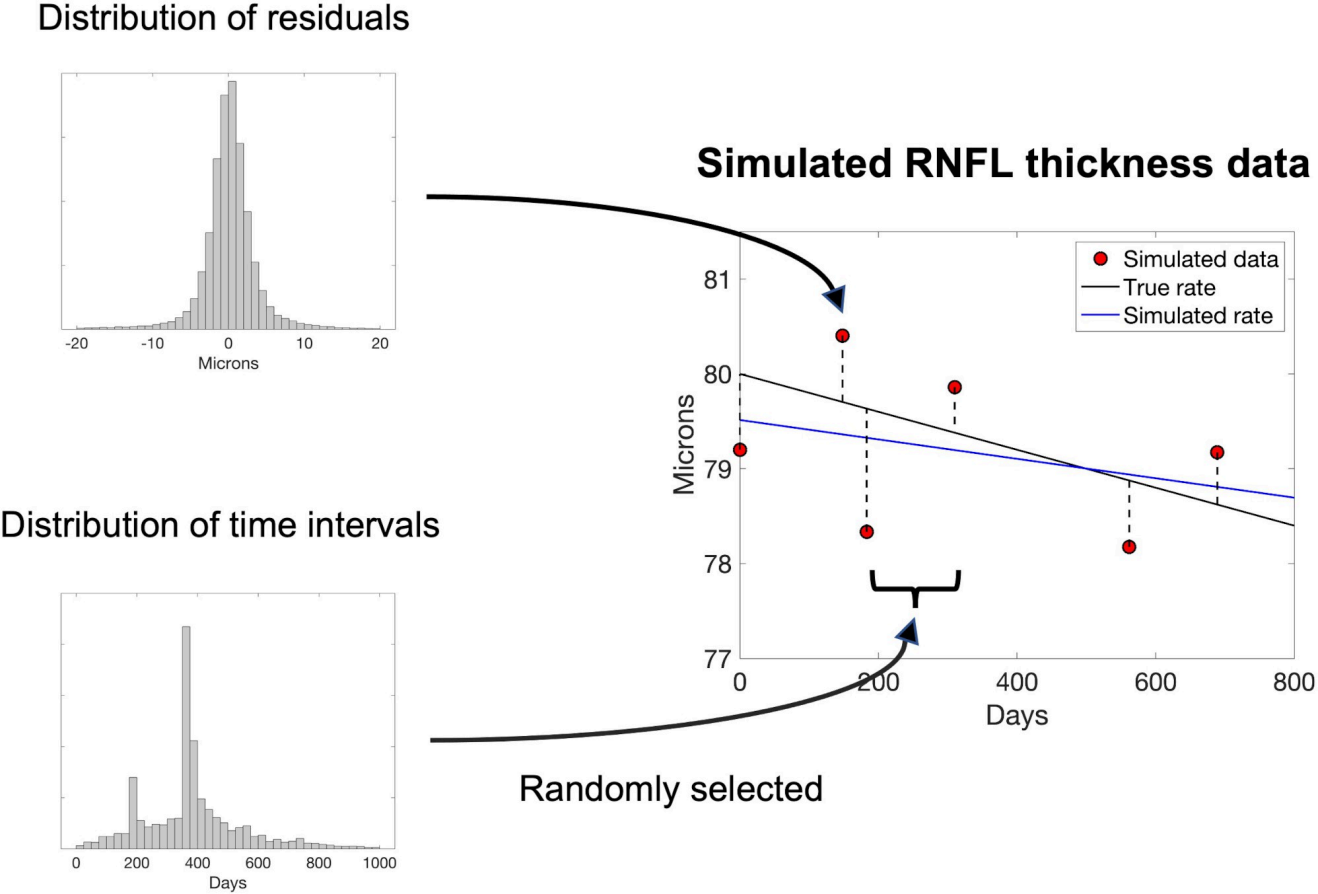
Schematic of how simulated RNFL thickness data points (red dots) were generated. Time intervals between simulated data points were randomly sampled from the distribution of time intervals between consecutive measurements in our sample. RNFL thickness was simulated at each time point by randomly selecting a residual from the combined distribution of residuals post-linear regression and adding it to the line that represented the “true” (i.e., no measurement error) rate of worsening (black line). The simulated rate of worsening (blue line) was the linear regression line through the simulated data points.

We used simulations to estimate the number of OCT RNFL thickness measurements needed for linear regression to outperform the two null models given current OCT examination frequencies and various rates of RNFL change (see Fig 1 for a schematic). For the simulations, we assumed that a linear model accurately describes true (i.e., without measurement error) OCT RNFL worsening, since all 5 models we compared were linear models. Time intervals between simulated data points were randomly selected from the combined distribution of time intervals between consecutive measurements in our sample. The mean (SD) time interval between consecutive measurements in our sample was 393 (190) days—a rate consistent with current insurance coverage which reimburse 1–2 scans per year [16]—and the mean (SD) residual was 0 (5.05) microns. RNFL thickness was simulated by adding a randomly chosen residual from the combined distribution of residuals post-linear regression at each time point. We used the combined distribution of residuals rather than distributions specific to a rate of worsening, or specific to a time point post-baseline, because of the need to simulate a wide range of conditions—sample size might otherwise become an issue.

For each set of M simulated data points, the simulated M+1st data point was generated with no measurement error (no residual added to the line). For each M, 10,000 sets of simulated measurements of RNFL thickness were generated to estimate the rate of RNFL worsening at which MAE was equivalent for both linear regression and the null model—the point at which MAE was equivalent was the rate beyond which linear regression outperformed the null model.

## Results

### Baseline demographics, clinical and OCT characteristics

Table 2 shows information about the 3,520 patients and 6,471 eyes that met our 4 inclusion criteria. Mean (SD) age was 72.90 (11.21), 57.07% were female, most were either white (56.51%) or black (35.37%), and most eyes (82.18%) had mild glaucoma. The mean (SD) number of scans per eye was 6.40 (1.41), the mean (SD) days between scans was 393 (190) days, and mean (SD) follow-up duration was 5.80 (1.25) years.

**Table 2:** Demographics and OCT data.

**Sample size**	
Patients	3,520
Eyes	6,471
**Age**	
Mean (SD)	72.90 (11.21)
Median	73.93
Range	23.58, 101.64
**Gender, n (%)**	
Male	1,511 (42.93%)
Female	2,009 (57.07%)
**Race, n (%)**	
White	1,989 (56.51%)
Black	1,245 (35.37%)
Asian	156 (4.43%)
Other	114 (3.24%)
N/A	16 (0.45%)
**Severity, n (%)**	
Mild	5,318 (82.18%)
Moderate	740 (11.44%)
Severe	413 (6.38%)
**OCT statistics, mean (SD)**	
Scans per eye	6.40 (1.41)
Days between scans	393 (190)
Follow-up duration (years)	5.80 (1.25)
Signal strength	7.80 (1.38)

### Prediction errors for various models

**Fig 2:**
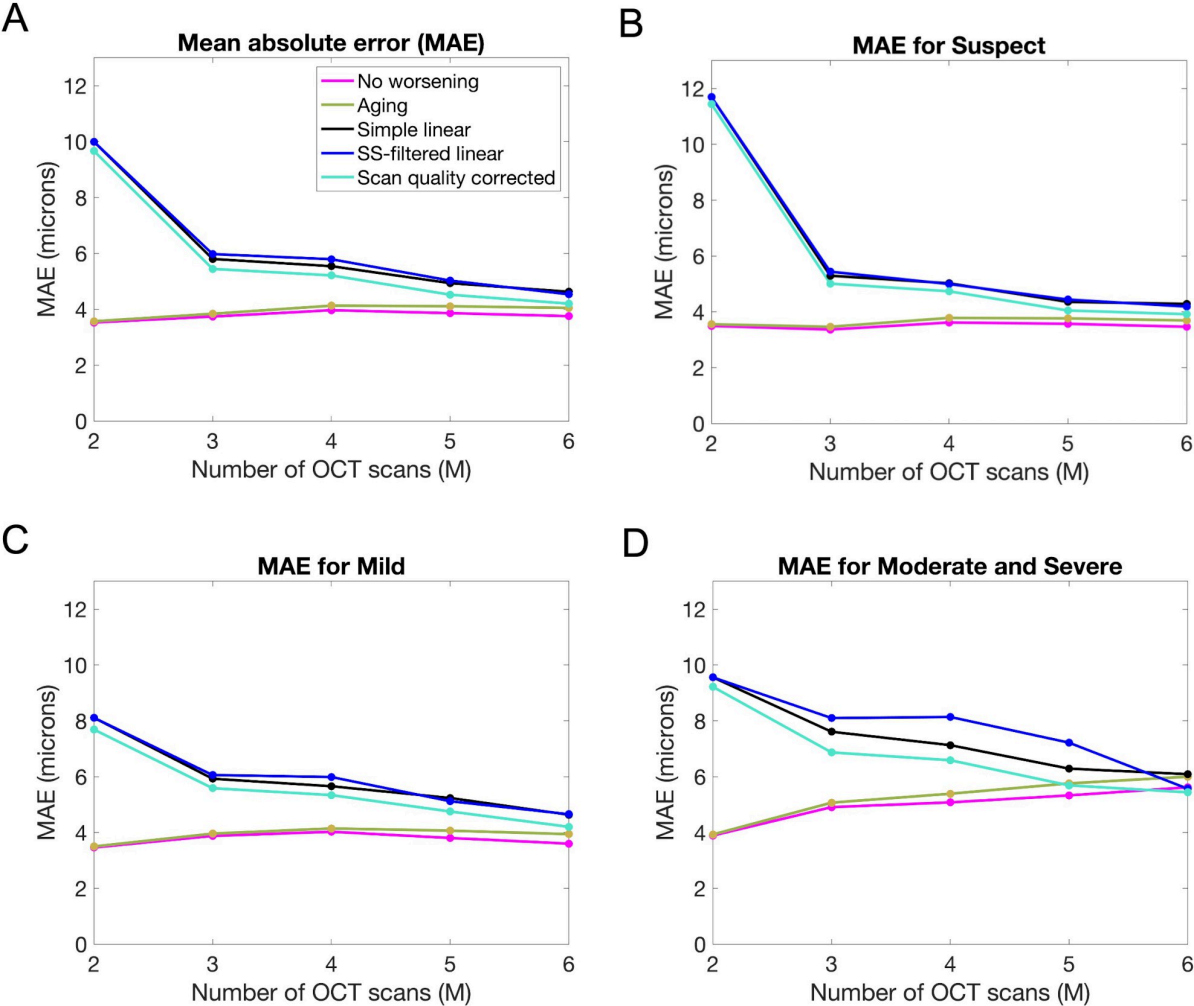
(**A**) MAE for different models predicting the M+1^st^ reliable measurement of RNFL thickness. Comparable results for (**B**) suspect, (**C**) mild, and (**D**) moderate and severe glaucoma.

Fig 2A shows that both null models had similar MAE, and all three linear models had similar MAE. Both null models outperformed all three linear models in predicting a reliable M+1^st^ measurement of OCT RNFL thickness up to the maximum of M = 9 tested (results are shown for 2 ≤ M ≤ 6 because of smaller sample sizes for M ≥ 7). As M increased, the difference in MAE between the null models and the other models decreased. The Wilcoxon rank sum test showed that the difference in distributions of absolute residuals for both null models compared to the simple linear and SS-filtered models was statistically significant for 2 ≤ M ≤ 6 with a p-value < 10^−5^ in all cases. Scan quality correcting RNFL thickness measurements prior to linear regression reduced this range to 2 ≤ M ≤ 5.

Fig 2B–2D show the effect of glaucoma severity on MAE, with moderate and severe categories combined due to small sample size. As severity increased, MAE increased for all models (for M ≥ 4), and the effect of scan quality correction became larger. The range of M for which differences in distributions of absolute residuals were statistically significant remained identical for suspect (Fig 2B) and mild (Fig 2C) glaucoma, compared to all data combined (Fig 2A). However, for moderate and severe glaucoma (Fig 2D), statistical significance was only achieved for 2 ≤ M ≤ 4 for both linear and scan quality corrected models, while the range was slightly larger at 2 ≤ M ≤ 5 for the SS-filtered model.

One reason both null models outperformed the linear models is because the equation of the best fitting line to all SS ≥ 8 reliable OCT measurements was *y* = -0.203*t* + 81.16, where *t* is time in years and *y* is RNFL thickness in microns. The 95% CI of the slope was (–0.290, –0.116), which lies between the predictions of the “no worsening” and “aging” models (0 and –0.365 μm/yr, respectively). In other words, the rate of change for SS ≥ 8 reliable OCT RNFL thickness measurements was close to the predictions of both “no worsening” and “aging” null models. In contrast, the best fitting line to the subset of reliable OCT RNFL thickness measurements from moderate or severe eyes was *y* = -0.475*t* + 71.38, which helps explain why MAE for the null models was greater for moderate and severe (Fig 2D).

### Results of simulations

**Fig 3:**
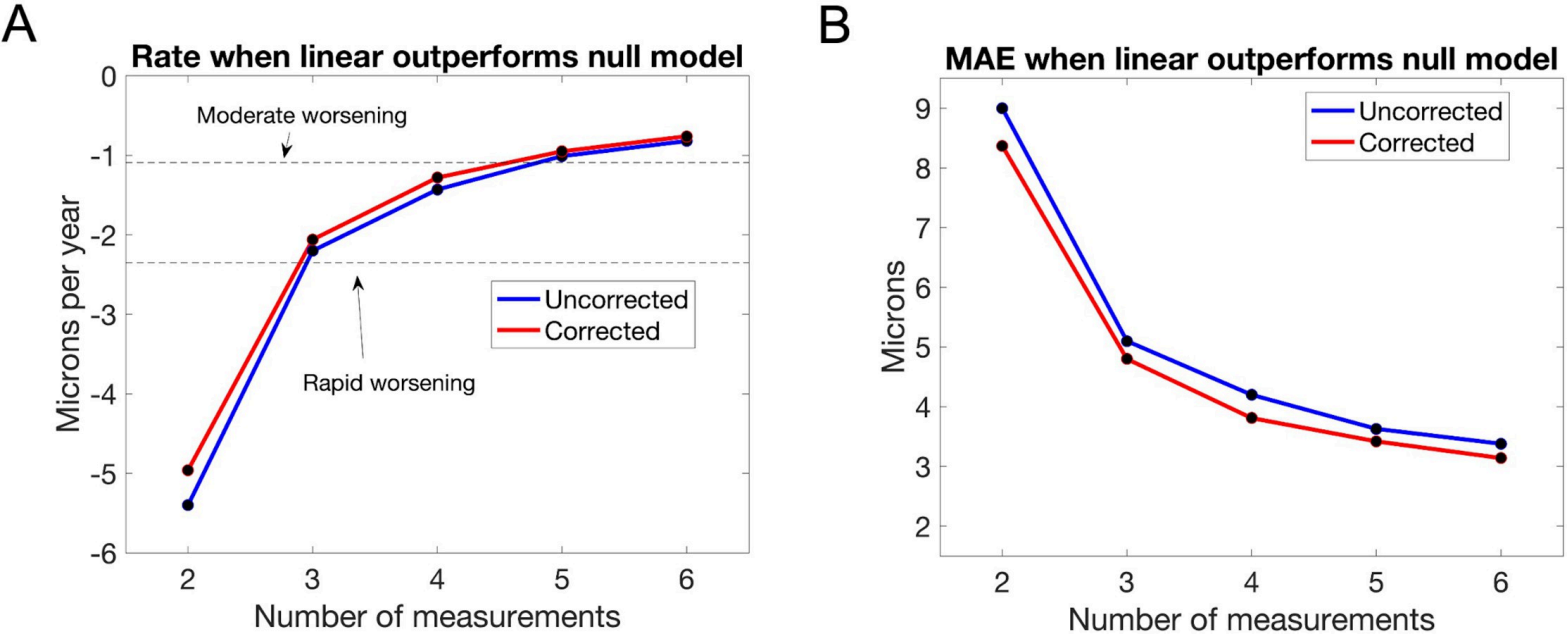
(**A**) The rate of worsening in μm/yr at which linear regression outperforms the “no worsening” null model for different numbers of simulated OCT RNFL thickness measurements (blue), as well as for different numbers of simulated scan quality corrected measurements of RNFL thickness (red), for OCT examination frequencies in our sample; and (**B**) the corresponding MAE. Dashed lines show the 75^th^ percentile (moderate) and 90^th^ percentile (rapid) rates of worsening measured in a previous study [10]. Results for the “aging” null model were virtually identical to the “no worsening” null model and are not shown.

Fig 3A shows the rate of worsening at which linear regression outperforms the “no worsening” null model for moderate (75^th^ percentile) and rapid (90^th^ percentile) RNFL worsening given OCT examination frequencies in our sample—results for the “aging” null model were virtually identical and are not shown. From a previous study, moderate worsening for a large glaucoma or glaucoma suspect population is –1.09 μm/yr, while rapid worsening is –2.35 μm/yr. At least 5 measurements of RNFL thickness are needed for linear regression to outperform the “no worsening” model for moderate worsening (the rates at 4 and 5 measurements are –1.43 μm/yr and –1.01 μm/yr, respectively), and at least 3 measurements are needed for rapid worsening (the rates at 2 and 3 measurements are –5.40 μm/yr and –2.20 μm/yr, respectively). Scan quality correction improved the rates (e.g., –4.96 μm/yr for 2 measurements instead of –5.40 μm/yr without scan quality correction) as well as the MAE, shown in Fig 3B. However, the number of measurements needed to outperform the null models remained the same as without scan quality correction.

## Discussion

This study shows that linear regression on OCT RNFL thickness measurements is less accurate at predicting future disease course in glaucoma than a null model that predicts no worsening, as well as a null model that predicts worsening due to aging alone, when using a small number of OCT scans obtained over time (M ≤ 6). Performance improves (M ≤ 5) when scan quality correction is applied to OCT RNFL thickness measurements prior to linear regression. Performance also improves when the goal is to detect more negative rates of RNFL thickness loss, i.e., further away from both null models. For example, at least 5 measurements are needed to outperform both null models for moderate worsening while only 3 measurements are needed for rapid worsening.

There are at least two reasons why both “no worsening” and “aging” null models outperformed linear regression with a small number of OCT scans. First, the –0.203 μm/yr slope of the best-fitting line to reliable OCT measurements in our sample is close to both zero (no worsening) and –0.365 μm/yr (aging). Second, the standard deviation of the residual distribution is comparatively high at 5.05 μm. With a small number of measurements, such a large standard deviation in the residual distribution means that there is a large amount of variability in the estimated slope of a linear model, which reduces its ability to detect smaller rates of change.

While the performance of linear models improves the greater the rate of RNFL thickness loss, it is a priori unknown which eyes will worsen faster. This means that in clinical practice, linear regression should be used to predict future disease course only when a sufficient number of OCT scans are obtained: 7 or more for suspect or mild, and 5 or more for moderate or severe, assuming one OCT scan per visit. These recommendations change if a more efficient measurement strategy is used, such as clustering, where half the total number of OCT scans are obtained at the beginning of the time period, and half at the end—there are theoretical reasons why this strategy should be near maximally efficient for linear regression [17]. With clustering, the minimum number of OCT scans reduces to 5 for suspect, 6 for mild and 4 for moderate and severe, assuming no scan quality correction. With scan quality correction, only 5 OCT scans are needed for mild. However, we note that implementing this clustering strategy in clinical practice would require knowing a priori how many total visits a patient will have.

Our results show that OCT scan quality correction based on disease severity and signal strength only slightly improves prediction of future reliable RNFL thickness measurements, though with greater disease severity the effect of correction is greater. As the implementation of such correction has a low computational burden, changes in OCT software that “correct” for errors are likely to improve the ability to detect RNFL change using linear regression. Other factors such as artifacts in the OCT scan and differences in retinal layer segmentation algorithms are likely important in explaining variability of RNFL measurements and OCT. Improvements in accounting for artifact and segmentation errors may also improve performance of the linear model. More complex machine learning approaches that incorporate these other sources of variability may also improve OCT scan quality correction.

The results of this study caution against relying on linear regression to forecast future disease trajectory with a small number of OCT scans. A previous study showed that even with a larger number of measurements, the accuracy of detecting glaucoma worsening may still be low (in many cases <50%) [10]. Detecting glaucoma worsening is different from forecasting future disease trajectory. Nevertheless, whether estimating the accuracy of detecting glaucoma worsening, or estimating the number of OCT scans needed for linear regression to more accurately forecast future disease trajectory, the recommendation is the same: OCT examination frequencies need to be increased beyond the typical 1–2 scans per year.

A major strength of this study is the large sample of glaucoma and glaucoma-suspect eyes treated in a clinical population. Our results are likely to apply to other treated populations. However, our results may not be generalizable to untreated populations. A major limitation of the study is that the “reliable” M+1^st^ measurement was not perfectly reliable. However, the mean of the combined distribution of residuals post-linear regression (Fig 3B) was almost exactly zero, which suggests that on average, the M+1^st^ measurement was not biased in a certain direction. Furthermore, the M+1^st^ measurement was perfectly reliable in our simulations.

Overall, our results show that, when relying on a small number of OCT scans over time as is often the case in clinical practice, linear regression of OCT RNFL thickness measurements using Zeiss Cirrus OCT is less accurate at predicting future reliable measurements than using the mean of all prior measurements (or assuming RNFL thickness loss due to aging alone). If clinicians aim to accurately predict future RNFL worsening over shorter periods of time (i.e., 1–2 years), more frequent OCT scans are needed to increase accuracy. Given that OCT scans are quick and relatively patient friendly, it may be practical to increase the number of tests to improve diagnostic accuracy.

## Supporting information

S1 Checklist *PLOS ONE* clinical studies checklist. (DOCX)
